# PReS-FINAL-2240: Serum amyloid protein a concentration in CAPS patients treated with anti IL1Β

**DOI:** 10.1186/1546-0096-11-S2-P230

**Published:** 2013-12-05

**Authors:** S Pastore, G Paloni, M Gattorno, A Taddio, L Lepore

**Affiliations:** 1 University of Trieste, Trieste, Italy; 2 UO Pediatria II, G. Gaslini Institute, Genoa, Italy; 3 University of Trieste, Institute for Maternal and Child Health - IRCCS "Burlo Garofolo, Italy; 4 Institute for Maternal and Child Health - IRCCS "Burlo Garofolo", Trieste, Italy

## Introduction

Although all patients with chronic inflammatory conditions are at risk for developing type AA amyloidosis, the incidence varies widely between the different Autoinflammatory syndromes. The reported incidence is about 35% of patients with CAPS (cryopyrin-associated periodic syndrome) which comprises familial cold auto-inflammatory syndrome (FCAS), Muckle-Wells syndrome and CINCA (chronic infantile neurological cutaneous, articular inflammatory syndrome). CAPS is associated with mutations in NLRP3/CIAS1 on chromosome 1q44, found only in about 50% of patients. Previous studies have demonstrated that IL-1β inhibitors are able to induce complete remission of clinical manifestations and suppression of markers of inflammation in the majority of patients.

## Objectives

To evaluate Serum Amyloid A (SAA) level in CAPS patients treated with anti IL-1β therapy and to correlate its level with the response to the treatment and with the presence of the NLRP3 mutation.

## Methods

We considered all patients of CAPS Italian Register affected by MWS or CINCA treated with IL-1β inhibitors (Anakinra or Canakinumab). According to Lachmann criteria a complete response to treatment was defined as a global assessment of no or minimal disease activity by a physician, an assessment of no or minimal rash, and a value for both serum CRP and SAA that was within the normal range (<0.5 mg/dL for CRP, < 6.4 mg/L for SAA). Partial response to IL-1β inhibitors was defined as a global assessment of no or minimal disease activity by a physician, and persistent elevated inflammatory markers (CRP, SAA). All patients underwent genetic analysis to identify NLRP3 mutations.

## Results

26 patients (15 M, 11 F) were considered, aged 2 to 52 years (median 16.5 ys). The mean duration of follow-up was 40 months. 18/26 patients were in treatment with Canakinumab (5 patients ab initio and 13 after a period with Anakinra therapy) and 8/26 patients were still taking Anakinra. 10/26 patients showed clinical remission with normal lab tests, including SAA. 10/26 patients presented clinical remission and normal CRP, but elevated SAA, with median value of 12.8 mg/L (IQR 10.5-16.8). 3/26 showed clinical remission but high values of SAA and CRP; 3/26 patients presented clinical remission, normal SAA values, but high CRP values. So in this series only 10/26 patients (38%) affected by CAPS showed a complete response to IL-1β inhibitors. In 18/26 patients we detected a mutation of NLRP3 gene. Median value of SAA in the mutated patients was 6,7 (IQR 2,3-13,3), median value in no mutated patients was 6,6 (IQR 4,1-46,6).

## Conclusion

In the previous studies complete response to anti-IL1 therapy fluctuates between 65% and 85%. By contrast, in our experience anti-IL1 inhibitors induced complete remission in only 38% of the patients because SAA values remained high (double value respect normal range) in half patients with no correlation between SAA values and genetic background. We do not know the real risk of these patients of developing amyloidosis, but we think that these patients need a constant long term follow up.

## Disclosure of interest

None declared.

